# Local and Systemic IL-7 Concentration in Gastrointestinal-Tract Cancers

**DOI:** 10.3390/medicina55060262

**Published:** 2019-06-10

**Authors:** Iwona Bednarz-Misa, Dorota Diakowska, Małgorzata Krzystek-Korpacka

**Affiliations:** 1 Department of Medical Biochemistry, Wroclaw Medical University, 50-368 Wroclaw, Poland; iwona.bednarz-misa@umed.wroc.pl; 2 Department of Gastrointestinal and General Surgery, Wroclaw Medical University, 50-368 Wroclaw, Poland; dorota.diakowska@umed.wroc.pl; 3 Department of Nervous System Diseases, Wroclaw Medical University, 51-618 Wroclaw, Poland

**Keywords:** interleukin IL-7, colorectal cancer, gastric cancer, esophageal cancer, lymph-node metastasis, immunotherapy for cancer, tumor microenvironment

## Abstract

*Background and objectives:* Interleukin-7 (IL-7) is exploited in cancer immunotherapies although its status in solid tumors is largely unknown. We aimed to determine its systemic and local concentrations in esophageal (EC), gastric (GC), and colorectal (CRC) cancers. *Materials and Methods:* IL-7 was immunoenzymatically measured in paired surgical specimens of tumors and tumor-adjacent tissue (*n* = 48), and in the sera of 170 individuals (54 controls and 116 cancer patients). *Results:* IL-7 was higher in tumors as compared to noncancerous tissue in all cancers (mean difference: 29.5 pg/g). The expression ratio (tumor to normal) was 4.4-fold in GC, 2.2-fold in EC, and 1.7-fold in CRC. However, when absolute concentrations were compared, the highest IL-7 concentrations were in CRC, both when tumor and noncancerous tissue were analyzed. In CRC tumors, IL-7 was 2 and 1.5 times higher than in EC and GC tumors. In noncancerous CRC tissue, IL-7 was 2.3- and 2.8-fold higher than in EC and GC. IL-7 overexpression was more pronounced in Stage 3/4 and N1 cancers as a result of decreased cytokine expression in noncancerous tissue. Tumor location was a key factor in determining both local and systemic IL-7 concentrations. Serum IL-7 in CRC and EC was higher than in controls, GC, and patients with adenocarcinoma of gastric cardia (CC), but no significant correlation with the disease advancement could be observed. *Conclusions:* IL-7 protein is overexpressed in EC, GC, and CRC, but concentrations differ both in tumor and tumor-adjacent tissue with respect to tumor location. More advanced cancers have lower IL-7 concentrations in the immediate environment of the tumor. At the systemic level, IL-7 is elevated in CRC and EC, but not CC or GC. IL-7 dependence on the location of the primary tumor should be taken into account in future IL-7-based immunotherapies. Functional studies explaining a role of IL-7 in gastrointestinal cancers are needed.

## 1. Introduction

Cancers of the gastrointestinal tract are the most frequent global malignancies. They include, but are not limited to, esophageal cancer (EC), gastric cancer (GC), and colorectal cancer (CRC). CRC, the most common of the gastrointestinal cancers, ranks fourth in incidence and second in mortality in both sexes combined according to GLOBOCAN 2018 estimates [1]. Although gradually decreasing worldwide, its incidence remains high in Europe. GC ranks fifth in incidence, but second in mortality, and remains the leading cause of infection-associated cancer deaths. The incidence rates of GC and CRC, especially those of rectal cancer, are particularly high in Eastern Europe. EC is less common, as it ranks seventh in incidence but is the sixth cause of cancer deaths [1]. Curative resection, in combination with chemo- or radiation therapy, remains the mainstay in the treatment of gastrointestinal-tract malignancies. Unfortunately, it could not improve the outcome in patients with metastatic cancers and/or cancers resistant to chemo- and radiotherapy. Therefore, new therapeutic approaches are searched for, among which immunotherapy is viewed as an attractive alternative that is gaining increasing interest [2]. Immunotherapy is meant to stimulate the host immune system to defeat cancer, and is considered to be more precise, potentially better-tolerated, and capable of exerting more durable responses than conventional chemotherapy [3]. Cytokines are crucial mediators of inflammatory and immune responses, potentially involved in cancer development and progression, as well as in the host defense against it. As such, both targeting the cytokine network and cytokine transfer are attractive options to be exploited for cancer immunotherapy [2,4]. However, a better understanding of cytokine-mediated tumor–cell interactions in the tumor environment is a prerequisite for developing precise and efficient immunotherapies.

Interleukin (IL)-7 is among the cytokines exploited as immunomodulators in cancer immunotherapy [4,5]. It was listed as one of the “Top Agents with High Potential for Use in Treating Cancer” [6]. This pleiotropic cytokine is crucial for survival, homeostasis, and the functioning of lymphocytes T and B [4]. Accordingly, its application in immune reconstitution in patients undergoing chemotherapy has been proposed [7]. IL-7 as an immunotherapeutic for cancer is particularly appealing since this cytokine does not induce hyperinflammation [8]. However, for such a renowned cytokine, surprisingly little is known on IL-7 in solid tumors. In fact, contradicting a beneficial role attributed to the cytokine, several reports have demonstrated IL-7 overexpression [9,10,11,12,13,14] and oversecretion [15,16,17,18,19,20,21,22] in cancer, and have linked it with unfavorable characteristics [9,12]. In light of the existing controversies, a need for further research on IL-7 has recently been stressed [23]. Our group was first to report systemic IL-7 elevation in CRC in association with metastatic disease and tumor location, as well as cytokine association with bowel inflammation [21]. However, supporting its auspicious character, we also demonstrated that the early postoperative rise in IL-7 is associated with favorable outcomes, such as lower incidence of surgical-site infections, milder surgery-induced lymphopenia, and beneficial interferon-γ dynamics [24]. Unlike other cytokines, IL-7 is primarily tissue-derived [25]. Therefore, in this study, we mainly aimed at determining local IL-7 concentrations in EC, GC, and CRC, and their association with tumor location, histology, and disease advancement. Additionally, we intended to compare systemic concentrations of the interleukin between cancer patients with tumors located in various parts of the gastrointestinal tract.

## 2. Materials and Methods

### 2.1. Study Population

The study population for the determination of local IL-7 concentrations consisted of 48 individuals with histologically confirmed cancers of the gastrointestinal tract, admitted to the Department of Gastrointestinal and General Surgery of Wroclaw Medical University for tumor curative resection. Patients suffering from any severe systemic illness, subjected to prior radio- or chemotherapy, or with a gross metastatic disease were excluded from the study. Patients were subjected to standard preoperative evaluation (blood work, physical examination, and imaging techniques such as ultrasonography, computed tomography, and magnetic resonance). Cancers were staged pathologically using the 7th edition of the Union for International Cancer Control (UICC) TNM system (tumor-node-metastasis) [26], and resection margins were confirmed to be tumor-free.

Study population for the determination of systemic IL-7 concentrations consisted of 170 individuals: 116 cancer patients and 54 healthy controls. Cancer patients were admitted to the Department of Gastrointestinal and General Surgery of Wroclaw Medical University for disease diagnosis and/or treatment, including curative surgery and palliative treatment. All had histologically confirmed carcinomas: squamous cell carcinomas of esophagus (EC) and adenocarcinomas of the gastric cardia (CC) or the stomach (GC). Blood samples were taken prior to any treatment. Cancers were staged clinically using the 7th edition of the UICC TNM system [26]. Controls were recruited from healthy volunteers from hospital staff, healthy outpatients of the Research, Science, and Educational Center of Dementia Diseases, Scinawa, Poland, suffering from headaches (dementia, neurological disorders, and brain tumors were excluded during diagnostic process), or from blood donors from the Regional Center of Blood Donation and Therapeutics in Wroclaw, Poland. 

### 2.2. Ethical Considerations

The study protocol was approved by the Medical Ethics Committee of Wroclaw Medical University (signature number: KB 203/2016). The study was conducted in accordance with the Helsinki Declaration of 1975, as revised in 1983, and informed consent was obtained from all patients.

### 2.3. Analytical Methods

Surgical tissue specimens were postoperatively collected from the tumor and from the macroscopically normal tissue taken approximately 10 cm from the tumor, rinsed with PBS, and kept frozen at −45 °C until examination.

Prior to analysis, tissue samples were weighed, and 10–40 mg fragments were homogenized (2 min, 4.0 m/s) in 10 mM Tris-hydrochloric acid with 150 mM potassium chloride and 1 mM ethylenodiamine tetraacetic acid (EDTA), pH 7.4 buffer (proportion 1:2 *w/v*), using a FastPrep-24 homogenizer and ceramic spheres (MP Biomedical, Solon, OH, USA). The resulting homogenates were clarified by centrifugation at 14,500× *g*, 10 min, 6 °C, and the supernatants were used for IL-7 determination.

Peripheral blood was collected upon admission prior to any treatment into BD Vacutainer CAT tubes (Becton Dickinson, Plymouth, UK) following an overnight fast, clotted (30 min, room temperature), and centrifuged (1500× *g*, 10 min, room temperature). Obtained sera were aliquoted and stored at −45 °C until examination.

IL-7 concentrations in tissue homogenates and paired serum samples were determined immunoenzymatically using Quantikine high-sensitive (HS) ELISA assays for human IL-7 from RnD Systems (Minneapolis, MN, USA) according to manufacturers’ instructions. All measurements were conducted in duplicates. Data are expressed as pg/mL of serum or pg/g of analyzed tissue.

IL-7 concentrations for systemic IL-7 analysis were determined using immunofluorescence and the BioPlex 200 platform with HRF (Bio-Rad, Hercules, CA, USA), incorporating Luminex xMAP^®^ technology, and Bio-Plex Pro™ Human Cytokine, Chemokine, and Growth Factor Magnetic Bead-Based Assays according to manufacturer’s instructions (Bio-Rad, Hercules, CA, USA). The method is based on flow cytometry and it utilizes magnetic microspheres conjugated with monoclonal antibodies. Standard curves were drawn using 5-PL logistic regression and the data were analyzed using BioPlex Manager 6.0 software.

Data on systemic IL-7 concentrations in CRC patients, measured using the above mentioned Luminex xMAP^®^ technology, were retrieved from our earlier paper [21] and used for comparative purposes.

### 2.4. Statistical Analysis

Normality of distribution was tested using the Kolmogorov–Smirnov test, and the homogeneity of variances using Levene’s test. Data were log-transformed to obtain normality of distribution if necessary. Between-group differences were analyzed using 1-way ANOVA (for multigroup comparisons) with Student–Neuman–Keuls post hoc test, or *t*-test for independent samples with Welch correction in case of lack of homogeneity of variances. Analysis of the paired samples was conducted using the *t*-test for paired samples. Data are presented as means, or geometric means in the case of log-transformed data, and accompanied by 95% confidence interval (CI) around the mean. Frequency analysis was conducted using the *χ^2^* test. Correlation analysis was conducted using Spearman’s rank correlation test (ρ). Least-squares multiple linear regression (stepwise method) was applied to identify independent predictors of local IL-7 expression. Only the variables found to be significantly associated with IL-7 in univariate analysis were entered into the model. Variables were entered into the model if *p* < 0.05 and removed if *p* > 0.1. Analysis of covariance (ANCOVA) was used to coexamine the effect of tumor location and cancer stage on systemic IL-7 concentrations. All calculated probabilities were two-tailed, and *p*-values ≤ 0.05 were considered statistically significant. Statistical analysis was entirely conducted using MedCalc Statistical Software, version 18.11.3 (MedCalc Software bvba, Ostend, Belgium; https://www.medcalc.org; 2019).

## 3. Results

### 3.1. Patient Characteristics

Detailed characteristics of patients in whom tissue IL-7 was examined are presented in Table 1, and of those enrolled for serum evaluation in Table 2.

### 3.2. IL-7 Protein Content in GI Tumors—Effect of Tumor Location

**Figure 1:**
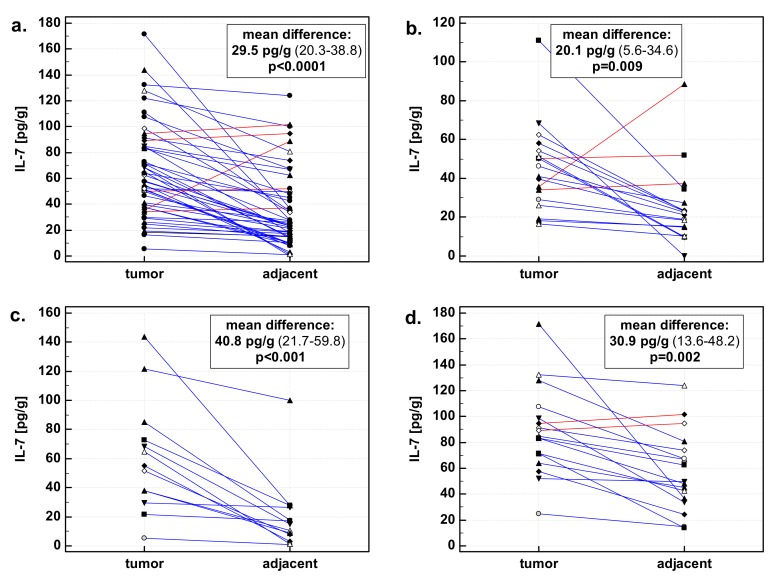
Interleukin-7 (IL-7) in tumor and patient-matched macroscopically normal tissue adjacent to the tumor: (**a**) All gastrointestinal cancers; (**b**) esophageal cancers; (**c**) gastric cancers; (**d**) colorectal cancers. Data expressed as picograms of IL-7 per gram of analyzed tissue. Data analyzed using *t*-test for paired samples. Presented in inserts, mean differences in IL-7 concentrations between tumor and patient-matched adjacent macroscopically normal tissue, together with 95% confidence interval around it and with statistical significance of difference. Cases with increased IL-7 concentration in the tumor are marked in blue, and cases with decreased IL-7 concentration in tumor in red.

In a whole cohort, the concentrations of IL-7 in tumors and patient-matched adjacent macroscopically normal tissue significantly differed. Mean IL-7 concentration in tumor tissue was 64.8 pg/g (95% CI: 54.3–75.4), while in adjacent normal tissue it was 35.3 pg/g (26.5–44.1). In a subgroup of patients with esophageal cancer, the mean difference was lower than the entire cohort average, but mean IL-7 concentration in tumors was higher than in normal tissue: 45.1 pg/g (33.9–56.3) vs. 25 pg/g (15.2–34.8). In a subgroup of patients with gastric cancer, the mean difference was higher than the entire cohort average. Mean IL-7 concentration in gastric tumors was higher than in normal tissue: 61.1 pg/g (37.7–84.6) vs. 20.4 pg/g (4.8–35.9). In a subgroup of patients with colorectal cancer, the mean difference was reflecting the whole cohort average. Mean IL-7 concentration in colorectal tumors was higher than in normal tissue: 88.6 pg/g (71.1–106) vs. 57.6 pg/g (41.8–73.5) (Figure 1).

**Figure 2:**
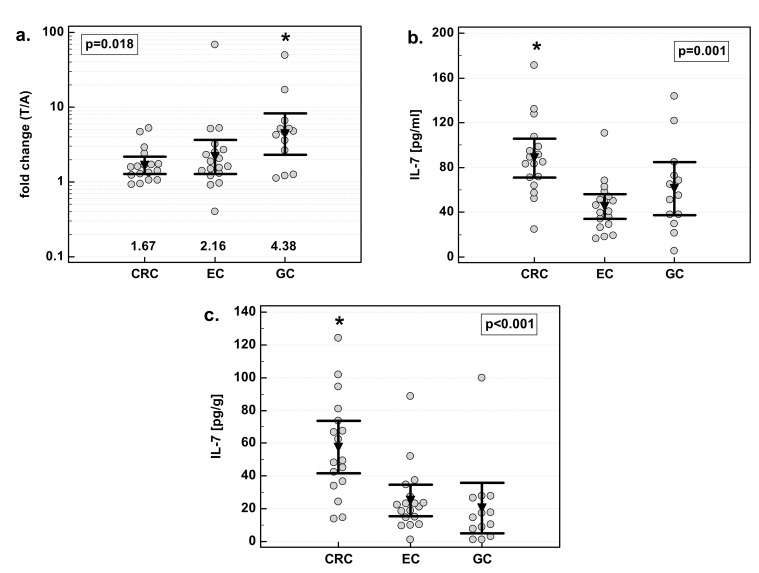
Effect of tumor location on IL-7: (**a**) fold change in IL-7 concentration; (**b**) IL-7 concentration in tumor tissue; (**c**) IL-7 concentration in adjacent macroscopically normal tissue. Data are expressed as picograms of IL-7 per gram of analyzed tissue. Data were analyzed with one-way ANOVA and presented as means with 95% confidence interval (triangle with whiskers). In case of data presented on the logarithmic scale, nontransformed values of geometric means are additionally given below the dot plots. T/A, tumor-to-adjacent tissue ratio; *, significantly different from other groups.

There was significant difference in fold change in IL-7 concentration with respect to tumor location—the highest increase was observed in gastric cancers. Colorectal cancers, in turn, had significantly higher concentration of IL-7 than other cancers, both when tumor and normal tissue were compared (Figure 2). As compared to EC and GC, CRC tumors had, respectively, two- and 1.5-fold higher concentration of IL-7. As compared to EC and GC, tumor-adjacent tissue from CRC patients had, respectively, 2.3- and 2.8-fold higher concentration of IL-7.

### 3.3. Effect of Tumor Histological Type on IL-7

**Figure 3:**
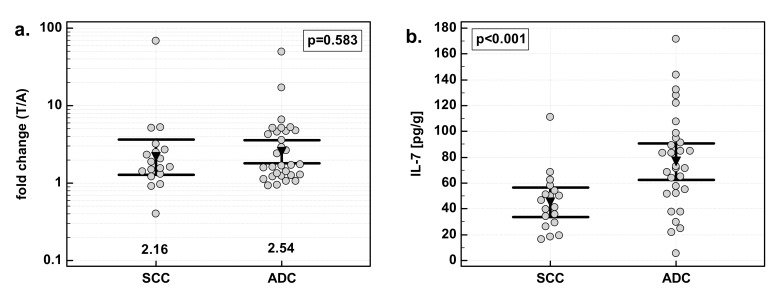

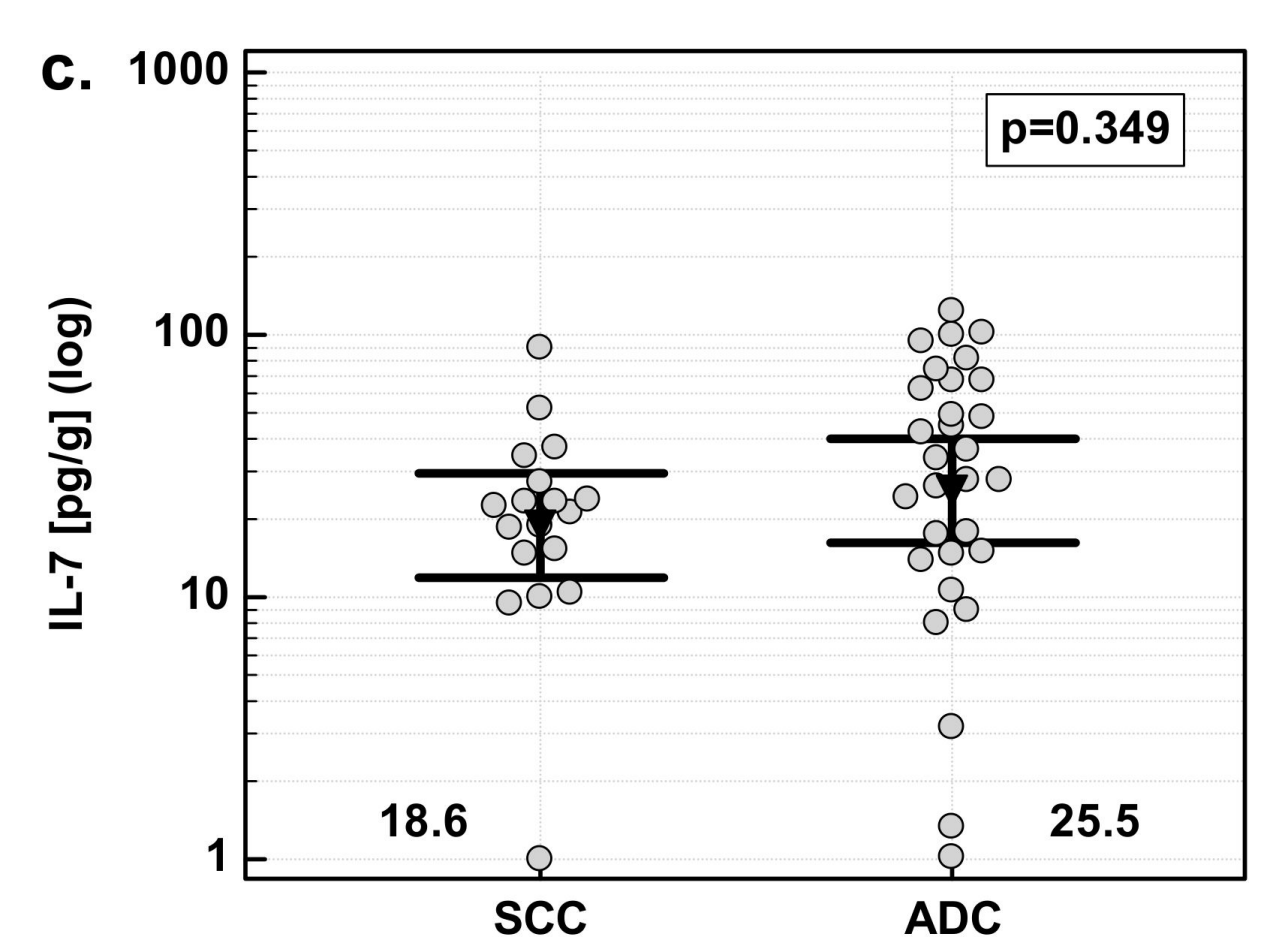
Effect of tumor histology on IL-7: (**a**) fold change in IL-7 concentration; (**b**) IL-7 concentration in tumor tissue; (**c**) IL-7 concentration in adjacent, macroscopically normal tissue. Data are expressed as picograms of IL-7 per gram of analyzed tissue. Data were analyzed with *t*-test for independent samples (tumors), or *t*-test with Welch correction (fold change and normal tissue) and presented as means with 95% confidence interval (triangle with whiskers). In case of data presented on the logarithmic scale, nontransformed values of geometric means are additionally given below the dot plots. T/A, tumor-to-adjacent tissue ratio.

There were no significant differences in IL-7 overexpression in tumor tissue with respect to tumor histology. Similarly, there was no difference in IL-7 concentration in macroscopically normal tissue adjacent to the tumor. However, there was significant difference in IL-7 concentration in tumor tissue. Adenocarcinomas (ADC) of the gastrointestinal tract had significantly higher IL-7 concentration in tumor tissue than squamous cell carcinomas (SCC) by 1.7-fold (Figure 3).

### 3.4. Effect of Cancer Stage on IL-7

**Figure 4:**
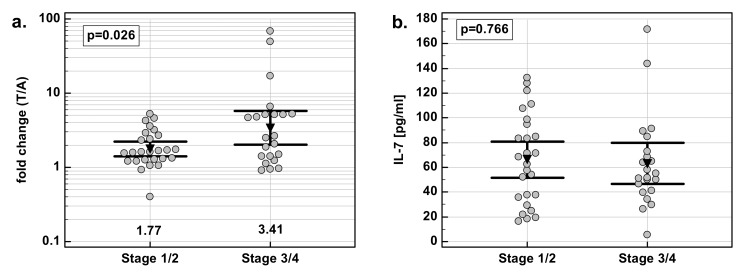

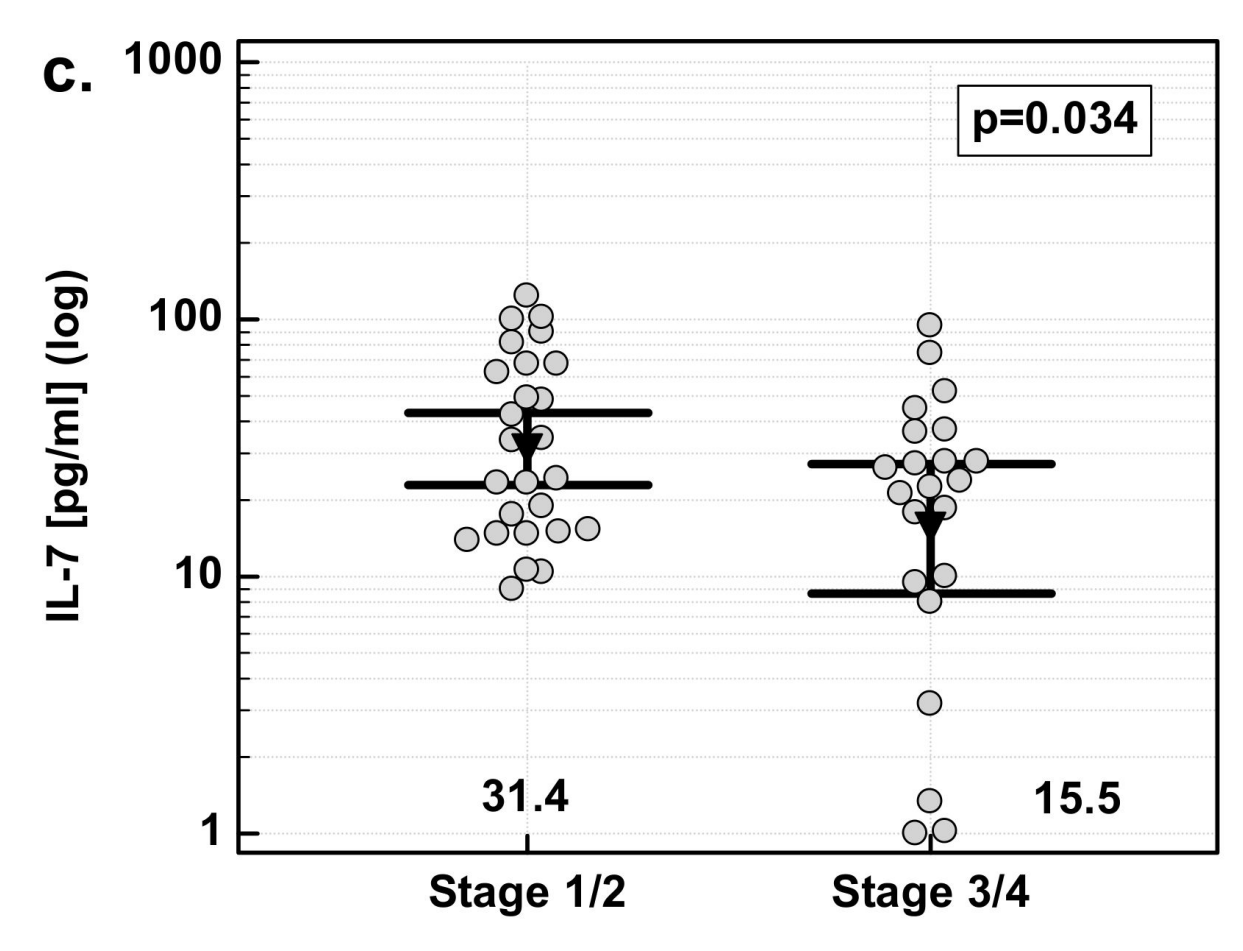
Effect of cancer stage on IL-7: (**a**) fold change in IL-7 concentration; (**b**) IL-7 concentration in tumor tissue; (**c**) IL-7 concentration in adjacent macroscopically normal tissue. Data are expressed as picograms of IL-7 per gram of analyzed tissue. Data were analyzed with *t*-test for independent samples (tumors), or *t*-test with Welch correction (fold-change and normal tissue), and presented as means with 95% confidence interval (triangle with whiskers). In case of data presented on the logarithmic scale, nontransformed values of geometric means are additionally given below the dot plots. T/A, tumor-to-adjacent tissue ratio.

There was significant difference in fold change in IL-7 concentration with respect to disease advancement. The overexpression of IL-7 in tumor tissue as compared to patient-matched normal tissue was significantly stronger in Stage 3/4 (T3/4) cancers than in less advanced Stage 1/2 (T1/2) cancers. The difference was caused by a significant difference in IL-7 concentration in macroscopically normal tumor-adjacent tissue: significantly higher in Stage 1/2 than Stage 3/4 cancers. IL-7 concentration in tumors did not differ with respect to the disease stage (Figure 4).

### 3.5. Effect of Primary Tumor Extent on IL-7

Overexpression of IL-7 in tumor tissue did not significantly differ with respect to the extent of the primary tumor. There was a 2.6-fold (95%CI: 1.8–3.9) increase in T3/4 tumors against a twofold (1.4–2.8) increase in T1/2 tumors (*p* = 0.286). Mean IL-7 concentration in T3/4 tumors did not differ from that in T1/2 tumors (respectively, 63.1 pg/g (49.5–76.6) and 68.4 pg/g (50–86.8), *p* = 0.638). In macroscopically normal tissue adjacent to T3/4 tumors, IL-7 concentration also did not significantly differ as compared to normal tissue adjacent to T1/2 tumors (respectively, 19.7 pg/g (12.6–30.9) and 30 pg/g (20–45.1); *p* = 0.223).

### 3.6. Effect of Lymph-Node Involvement on IL-7

**Figure 5:**
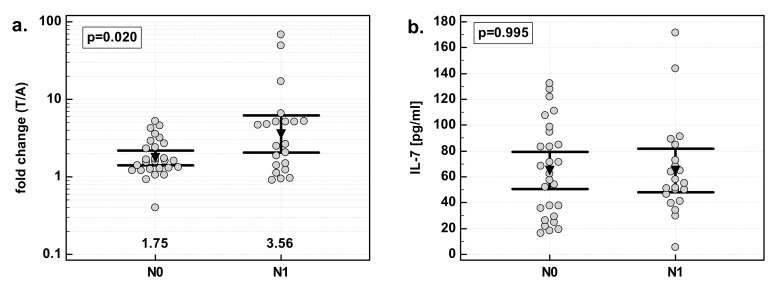

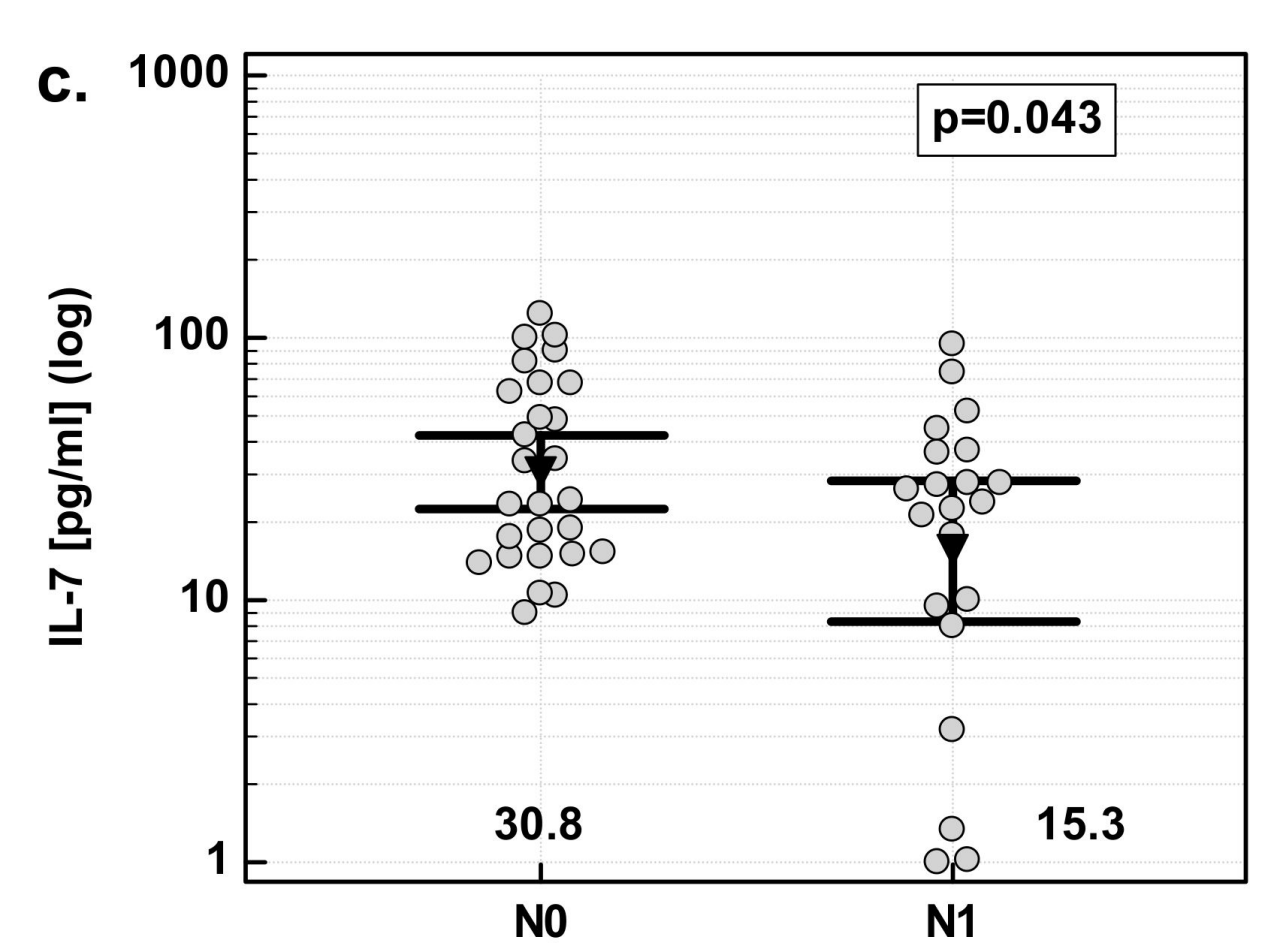
Effect of lymph-node metastasis on IL-7: (**a**) fold change in IL-7 concentration; (**b**) IL-7 concentration in tumor tissue; (**c**) IL-7 concentration in adjacent macroscopically normal tissue. Data are expressed as picograms of IL-7 per gram of analyzed tissue. Data were analyzed with *t*-test for independent samples (tumors), or *t*-test with Welch correction (fold-change and normal tissue), and presented as means with 95% confidence interval (triangle with whiskers). In case of data presented on the logarithmic scale, nontransformed values of geometric means are additionally given below the dot-plots. T/A, tumor-to-adjacent tissue ratio; N0, cancers without lymph-node metastasis; N1, cancers with lymph-node metastasis.

There were significant differences in fold change in IL-7 expression with respect to lymph-node involvement. The overexpression of IL-7 in tumor tissue as compared to patient-matched normal tissue was significantly stronger in patients with cancers with lymph-node metastasis (N1) than in those without (N0). Similar to the effect of the overall stage, the difference resulted from differences in normal tissue. IL-7 concentration was significantly higher in N0 than N1 cancers in macroscopically normal tissue adjacent to the tumor, while there was no difference in tumor tissue (Figure 5).

### 3.7. Effect of Distant Metastases on IL-7

There were no significant differences in IL-7 overexpression in tumor tissue as compared to patient-matched normal tissue with respect to the presence of distant metastases. Fold change in IL-7 concentration in M1 cancers was 3.1 (0.9–11.1) against 2.3 (1.8–3.1) in M0 cancers; *p* = 0.556. There was no significant difference in IL-7 concentration in the primary tumor between M1 and M0 cancers (respectively, 51.3 pg/g (23.2–79.3) and 66.1 pg/g (54.7–77.5); *p* = 0.440), or in tumor-adjacent macroscopically normal tissue (15.5 pg/g (5.5–43.8) and 23.5 pg/g (16.6–33.3); *p* = 0.481).

### 3.8. Independent Predictors of IL-7 Content (Multivariate Analysis)

To identify independent predictors of IL-7 overexpression in tumors, multiple regression (stepwise method) was applied. Variables found significantly associated with fold change in IL-7 concentration (dependent variable), that is, dichotomized tumor location (GC encoded as 1, and EC and CRC encoded as 0), dichotomized stage (Stages 1 and 2 encoded as 0, and Stages 3 and 4 encoded as 1), and lymph-node involvement were entered into analysis as explanatory variables. Of these, tumor location in the stomach alone was significantly associated with IL-7 overexpression, explaining 26% variability in IL-7 fold change. Partial correlation coefficient (r_p_) for the net association between IL-7 overexpression and tumor location in the stomach was 0.51. The relation of IL-7 overexpression with cancer stage and lymph-node involvement lost its significance when coexamined with tumor location (Table 3).

To identify independent predictors of IL-7 concentration in tumor tissue, tumor location (CRC encoded as 1, and EC and GC encoded as 0) and histology (ADC encoded as 1 and SCC as 0) were entered into analysis as explanatory variables. Tumor location in the colorectum was found independently associated with IL-7 concentration in tumor tissue, explaining 22% of variability in its variability (Table 3). 

To identify independent predictors of IL-7 concentration in macroscopically normal tissue adjacent to the tumor, tumor location (CRC encoded as 1 and EC and GC encoded as 0), stage, and lymph-node involvement were entered into analysis as explanatory variables. Tumor location in the colorectum was independently associated with IL-7 concentration in macroscopically normal tissue adjacent to the tumor, explaining 27% in its variability (Table 3).

### 3.9. Correlation between Local IL-7 Concentration and Its Serum Levels

Possible correlation between IL-7 overexpression, its concentration in tumor tissue and tumor-adjacent macroscopically normal tissue, and systemic concentrations of IL-7 were examined. Of those, IL-7 in serum correlated positively with IL-7 concentration in tumor tissue, but only in patients with adenocarcinomas (ρ = 0.45, *p* = 0.013).

### 3.10. Serum Concentrations of IL-7

**Figure 6:**
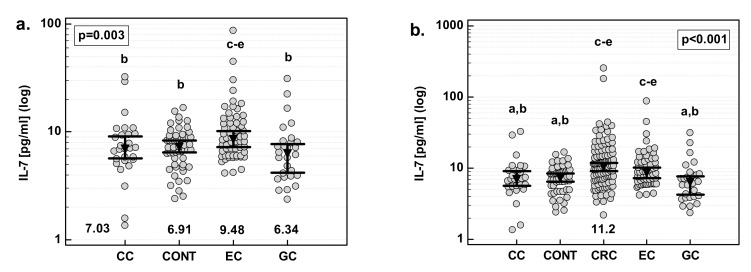
Serum concentrations of IL-7 (**a**) in a study population and (**b**) as compared with CRC. Data were analyzed with one-way ANOVA and are presented as means with 95% confidence interval (triangle with whiskers; numbers below dot plot). Letters above dot plots indicate significance of between-group differences: a, significantly different from CRC; b, significantly different from EC; c, significantly different from CC; d, significantly different from CONT; e, significantly different from GC. CC, cancer of the cardia.

EC patients had significantly higher IL-7 than healthy controls, and CC and GC patients. There were no statistical differences between CC and GC patients, and between those patients and healthy controls (Figure 6a). Additionally, for comparative purposes, we used data previously obtained for CRC patients [21]. CRC patients had the highest IL-7, significantly when compared to CC and GC but not EC patients (Figure 6b).

Except for the association of systemic IL-7 with tumor location in the gastrointestinal tract, there were no other significant correlations between serum interleukin concentrations and clinical features of the disease. Serum IL-7 was not correlated with cancer stage, the extension of the primary tumor, lymph-node metastasis, or distant metastasis.

However, there was a difference in disease-stage distribution between examined cancers. Therefore, multivariate analysis was done in which tumor location was entered as an explanatory variable and cancer T (*p* = 0.364), N (*p* = 0.533), and M (*p* = 0.865) were taken into account as possible covariates. Even with those differences accounted for, tumor location (*p* = 0.004) was significantly associated with systemic IL-7 concentrations.

## 4. Discussion

Unlike other interleukins, secreted primarily by immune cells, IL-7 is a tissue-based interleukin [25], yet reports on its expression in solid tumors are scanty. In fact, research on IL-7 focused on its function as a “critical enhancer of protective immunity” [27], and its potential contribution to cancer development had not entered the scene until recently. Still, it is surprising as, even with the assumed beneficial role of IL-7, extensive knowledge on cytokine status in cancers to be treated with it ought to be a prerequisite. IL-7 plays a pivotal role in immunity, and potentially confers protection against cancer via its effect on T lymphocytes. Accordingly, the antitumor activity of IL-7 has been shown in vitro and in vivo in animal models of prostate cancer, melanoma, and some neurological and blood cancers (reviewed in Reference [23]). However, some doubts have arisen about its positive role in cancer resulting from observations on upregulated expression [9,10,11,12,13,14] and/or secretion [15,16,17,18,19,20,21,22] in several cancers. Moreover, IL-7/IL-7R expression [10,12,28], as well as IL-7 secretion [15,19,20,21], have been correlated with adverse clinical outcomes in most, but not all [13,24], studies. These observations have been followed by functional studies demonstrating mitogenic [28] and antiapoptotic activity [29] of IL-7 toward lung-cancer cells and its ability to induce VEGF-D expression and promote lymphangiogenesis in lung- [28] and breast-cancer [30] models.

In this study, we demonstrated and quantified, for the first time, the expression of IL-7 at the protein level in tumors of the gastrointestinal tract with reference to disease advancement. We showed that IL-7 was overexpressed, to different degrees, by esophageal, gastric, and colorectal cancers. In addition to previously observed elevation of IL-7 at the systemic level in CRC [21], also esophageal cancer was associated with the oversecretion of IL-7. No such association was observed for cancers of the gastric cardia or the stomach.

Concerning colon cancer, IL-7 was evaluated in single-cell suspensions derived from patients [31], in human colon carcinoma xenografts [32], and mouse models of peritoneal and lung metastasis [33]. Maeurer et al. [31] demonstrated that tumor cells obtained from CRC patients synthesize and secrete IL-7, and that mRNA for IL-7 is present in both tumor and tumor-adjacent tissue. We, in turn, showed that these results translate into protein, as IL-7 was detectable in both tumors and macroscopically normal tissue adjacent to the tumors. Studies on the IL-7 effect on colon-cancer cells yielded consistent results, showing that IL-7 alone had neither a positive nor a negative effect on tumors. Unlike in breast and lung cancers, IL-7 did not stimulate proliferation of colon-cancer cells [31,33]. Of note, the lack of mitogenic activity toward keratinocytes was recently also observed [34]. On the contrary, in combination therapy with adoptively transferred human peripheral blood T cells, it significantly improved the survival of tumor-bearing animals [32]. In combination with oxaliplatin, in turn, it significantly enhanced the ability of oxaliplatin to inhibit proliferation and induce apoptosis in cancer cells [33]. In both studies, histological analysis showed increased tumor infiltration with activated CD8^+^ T cells [33] or their enriched population [32], and decreased number of regulatory T cells [33] in the spleen. Maurer et al. [31] also observed that IL-7 caused the expansion of tumor-infiltrating lymphocytes. However, Murphy at al. [32] showed that those lymphocytes had poor cytolytic activity, and that their antitumor effect was mediated by IFN-γ. This finding is in agreement with that made with regard to combination therapy with checkpoint inhibitors for bladder cancer [35], which demonstrated the interdependence of IL-7/IL-7R and IFN-γ/IFN-γR pathways, as well as their necessity for the immune checkpoint blockade to be effective. Of note, the interplay of both pathways is also reflected at the systemic level through close correlation between IL-7 and IFN-γ [21,24].

Corresponding with its protective role against infection [26], IL-7 concentrations in both normal and tumor tissue were the highest in the colon, a part of gastrointestinal tract with the highest exposure to bacteria. Indeed, cytokine concentrations in inflammatory bowel disease are higher than in CRC cancer [21]. Serum levels of CRC patients were also the highest in all the examined gastrointestinal cancers. However, the highest difference in IL-7 concentration between tumor and patient-matched normal tissue was in gastric cancer. Only recently, Greenstein et al. [22] published an abstract reporting elevated systemic IL-7 in gastric cancer. In our group, systemic IL-7 in GC was the lowest in the examined cancers and did not significantly differ as compared to the controls. Similarly, systemic levels of IL-7 in adenocarcinomas of the gastric cardia were at the levels observed in controls. While our observations relating to local IL-7 expression corroborate those of Greenstein [22], Quan et al. [36] listed IL-7 among 105 molecules, out of 507 examined, differently expressed in gastric tumors as compared to normal tissue. However, in their study, tumor IL-7 expression constituted 9% of that found in normal tissue. The discrepancy between studies might result from theirs being based on only eight patients, and on the much less sensitive and semiquantitative method of cytokine determination. Moreover, out of those 105 differently expressed molecules, only few have been upregulated in tumors—a highly improbable result. Like in bowel inflammation, the upregulation of IL-7 mRNA has been reported in association with gastritis in *Helicobacter pylori*-positive patients [37]. 

We also observed the IL-7 upregulation in EC tumors with a corresponding increase of its levels in sera of EC patients. Systemic concentrations of IL-7 were at the level observed in CRC [21]. To the best of our knowledge, these are first reports concerning IL-7 at the protein level in EC. Previous findings on IL-7 in EC are limited to Oka et al. [38] reporting on the cytokine being expressed by five out of six cell lines, and a recent report of Kim et al. [14] on cytokine receptor being overexpressed at mRNA level in patients with esophageal squamous cell carcinoma. These authors attributed the oncogenic role to IL-7R, and observed that the loss of its expression had an antitumor effect.

Tumor location was a key determinant of IL-7 expression in the current study, both at the local and systemic level. However, we also observed that IL-7 was locally overexpressed in gastrointestinal tumors from Stage 3/4 and N1 cancers to a greater degree than in tumors from Stage 1/2 and N0 cancers. This observation corroborates previous findings on IL-7 at the systemic level in CRC [15,19,21] and ovarian cancer [20]. Interestingly, the magnitude of overexpression observed here was twofold, exactly like in the case of ovarian cancer [20]. However, we failed to observe significant association with the disease advancement at the systemic level. This may result from most of the patients enrolled for serum analysis of IL-7 having advanced cancers, not amenable for curative resection. Thus, they were staged clinically, as a more accurate pathological evaluation was unavailable.

IL-7 overexpression observed here, as well as by others, may not necessarily be a causative factor for adverse effects. It has been related to some favorable outcomes as well—IL-7 overexpression correlated with better survival in patients with head and neck cancers [13], improved wound healing [34], milder surgery-induced lymphopenia, and lower incidence of surgical-site infections [24]. It also plays a crucial defensive role in colon infections with pathogenic micro-organisms [26]. Moreover, in line with the beneficial role proposed for IL-7, it has been suggested that IL-7 overexpression might be a compensatory mechanism to overcome hampered IL-7/IL-7R signaling [22]. Indeed, while Greenstein et al. [22] showed that IL-7 is a substrate for the MMP-9 protease, an enzyme known to be overexpressed in cancer [39], others demonstrated that T-cell responsiveness to IL-7 was obstructed by cell exposure to inflammatory cytokines [40]. Moreover, the upregulation of MMP-9 expression and activity is associated with tumor angiogenesis, invasion, and metastasis [39] when IL-7 is overexpressed as well. Furthermore, as shown by Greenstein et al. [22], andecaliximab, an antibody against MMP-9, restored IL-7 levels in gastric-cancer patients. Interestingly, our results showed that IL-7 overexpression in advanced cancers was not caused by increased cytokine concentration in tumors, but rather by its decreased concentration in noncancerous tissue adjacent to the tumor. This finding might be interpreted in the favor of beneficial role attributed to IL-7 and imply that the loss of IL-7 in the immediate environment of the tumor may facilitate cancer progression. Correspondingly, cancer-associated chronic inflammation creates an immunosuppressive microenvironment, facilitating disease development [41]. Decreased IL-7 concentration around the tumor might be an additional effect of the impaired IL-7/IL-7R signaling [22] mechanism of cancer-induced immunosuppression. 

## 5. Conclusions

The interleukin-7 protein is overexpressed in esophageal, gastric, and colorectal cancers, but only CRC and EC patients had significantly elevated IL-7 at the systemic level as compared to healthy controls. Its concentrations differ with respect to tumor location, both in tumor and tumor-adjacent tissue, as well as at the systemic level. More advanced cancers have lower interleukin-7 concentrations in the immediate environment of the tumor, which may constitute a mechanism of cancer-induced immunosuppression and facilitate disease progression. These observations might be relevant for future IL-7-based immunotherapies.

## Figures and Tables

**Table 1:** Characteristics of study population (local IL-7 concentrations).

Characteristics	EC	GC	CRC	*p*-Value
N	18	13	17	-
Sex (F/M), n	8/10	3/10	6/11	0.471 ^χ^2^^
Age (yrs.), mean ± SD	64.1 ± 5.6	68.2 ± 9.8	68.2 ± 11.5	0.331 ^A^
Histology	SCC	ADC	ADC	-
TNM ^1^ stage (1/2/3/4), n	2/6/9/1	2/3/5/3	5/8/4/0	0.147 ^χ^2^^
Tumor extension, T (1/2/3/4), n	1/6/9/2	1/1/9/2	1/6/10/0	0.495 ^χ^2^^
Lymph-node metastasis, N (0/1), n	9/9	5/8	13/4	0.092 ^χ^2^^
Distant metastases, M (0/1), n	17/1	10/3	17/0	0.066 ^χ^2^^

EC, esophageal cancer; GC, gastric cancer; CRC, colorectal cancer; N or n, number of observations; F/M, female-to-male ratio; yrs., years; SD, standard deviation; SCC, squamous cell carcinoma; ADC, adenocarcinoma; ^χ^2^^, Chi-square test; ^A^, 1-way ANOVA; ^1^, tumor-node-metastasis classification system of malignant tumors.

**Table 2:** Characteristics of study population (systemic IL-7 concentrations).

Characteristics	CONT	EC	CC	GC	P
N	54	60	28	28	-
Sex (F/M), n	23/31	20/40	7/21	9/19	0.427 ^χ^2^^
Age (yrs.), mean ± SD	65 ± 8	63 ± 9	63 ± 9	64 ± 10	0.237 ^A^
Histology	-	SCC	ADC	ADC	-
TNM ^1^ stage (1/2/3/4), n	-	6/11/15/28	0/2/4/22	1/5/5/17	0.132 ^χ^2^^
Tumor extension, T (1/2/3/4), n	-	6/10/14/30	0/1/3/24	1/2/9/16	0.030 ^χ^2^^
Lymph node metastasis, N (0/1), n	-	19/41	2/26	6/22	0.039 ^χ^2^^
Distant metastases, M (0/1), n	-	33/28	6/22	11/17	0.017 ^χ^2^^

CONT, healthy controls; BEN; individuals with benign diseases of the esophagus or gastric cardia; ^χ^2^^, Chi-square test; ^A^, 1-way ANOVA; ^1^, tumor-node-metastasis classification system of malignant tumors.

**Table 3:** Results of least-squares multiple regression.

Dependent Variable	Independent Variables	Regression Equation		Model Goodness of Fit (R^2^)	Analysis of Variance (F-Ratio, *p*)
		Constant, Coefficients *b*	t, p, r_p_		
Fold change in IL-7 concentration (log)	GC stage N	const.: 0.235	t = 3.94	0.257	F = 15.5, *p* = 0.0003
			*p* < 0.001		
		coeff.: 0.406			
			r_p_ = 0.51		
IL-7 concentration in tumor tissue (log)	CRC histology	const.: 1.636	t = 3.59	0.219	F = 12.9, *p* = 0.0008
			*p* < 0.001		
		coeff.: 0.278			
			r_p_ = 0.47		
IL-7 concentration in adjacent tissue (log)	CRC stage N	const.: 1.173	t = 4.1	0.268	F = 16.8, *p* = 0.0002
			*p* < 0.001		
		coeff.: 0.518			
			r_p_ = 0.52		

N, lymph node involvement; const., constant; coeff., coefficient; r_p_, partial correlation coefficient; R^2^, coefficient of determination. Independent variables retained in the model are in bold and underlined.
